# The efficacy of Qigong exercises for nonalcoholic fatty liver disease: A protocol for systematic review and meta-analysis

**DOI:** 10.1097/MD.0000000000022753

**Published:** 2020-10-30

**Authors:** Yuqiao Liu, Jiaxi Zou, Lijuan Dan, Renyan Zhang, Quansheng Feng

**Affiliations:** Chengdu University of Traditional Chinese Medicine, Chengdu, China.

**Keywords:** meta-analysis, nonalcoholic fatty liver disease, protocol, qigong, systematic review

## Abstract

**Background:**

Nonalcoholic fatty liver disease (NAFLD) is one of the most common chronic liver diseases in the world that represents an important public health challenge nowadays. Lifestyle changes and exercise can reduce the development of fatty liver. The aim of this systematic review and meta-analysis is to evaluate the treatment efficacy of Qigong for NAFLD.

**Methods:**

A detailed search for articles up to September 2020 will be performed to identify randomized controlled trials for Qigong in NAFLD. The following database will be used: PUBMED, Embase, Web of Science, Cochrane Library, Sino Med, Chinese National Knowledge Infrastructure (CNKI), Chinese Science and Technology Periodicals Database, and Wanfang Databas. Grey literature will be explored and the selection of studies, data extraction and validation will be performed independently by 2 reviewers using predefined selection criteria and quality indicators. Stata V.13.0 and Review manager 5.3 software will be used for data synthesis, sensitivity analysis, subgroup analysis, and risk of bias assessment. We will use the grading of recommendations assessment, development, and evaluation system to assess the quality of evidence.

**Results:**

This research will provide a quantitative and standardized assessment of the treatment efficacy of Qigong for NAFLD.

**Conclusion:**

This systematic review will generate the latest evidence for determining whether Qigong has a positive treatment effect for NAFLD.

**Registration number:**

INPLASY202090034

## 1 Introduction

Nonalcoholic fatty liver disease (NAFLD) has become the most common liver disease worldwide.^[1]^ It constitutes a spectrum ranging from simple steatosis through to nonalcoholic steatohepatitis (NASH) and cirrhosis of the liver. NASH can cause liver cirrhosis, liver failure, and liver cancer.^[2]^ The overall prevalence of NAFLD worldwide continues to increase due to the large-scale spread of obesity, especially in Western countries. And the pooled prevalence of NAFLD was 24.24%.^[3]^ Consequently, NAFLD has become a leading indication for liver transplantation. Although genetic factors play an important role in the pathogenesis of NAFLD, detrimental lifestyle, prolonged sedentary periods or limited physical activity have major metabolic implications. To date, the US Food and Drug Administration (FDA) has not approved any drugs to treat NASH. The cornerstone of the treatment of mild or nonadvanced forms NAFLD, is lifestyle changes, including modifications to diet and physical activity, to reduce body weight and liver fat.^[4]^

Qigong, a traditional fitness method that originates from ancient China. The practice of qigong aims to cultivate energy via systematic training exercises, including the coordination of different breathing patterns, rhythmic movements, and meditation, in contrast to conventional exercise.^[5]^ Qigong is appropriate for nearly anyone of any age or physical condition, due to its significant promotion of human health and ease of learning. The word “qigong” involves 2 theories: “qi,” the vital energy of the body, and “gong,” the training or cultivation of qi.^[6]^ Therefore, one of the possible explanations for the beneficial effects of Qigong exercise is increased healthy flow of qi, blood, and fluid throughout the body by repetitive movements to relieve pathological stagnation and regulate the function of meridians and visceral organs. Qigong is practiced by Chinese people to improve health, explore the latent ability of humans, prevent disease, and prolong life in the context of a wide range of conditions. In this study, we will evaluate the safety and efficacy of Qigong for NAFLD, so as to provide evidence for treating NAFLD.

## 2 Methods

### 2.1 Study registration

This meta-analysis was registered with the International Platform of Registered Systematic Review and Meta-Analysis Protocols (INPLASY) on 8 September, 2020 (registration number INPLASY202090034). The Preferred Reporting Items for Systematic Review and Meta-Analysis Protocols (PRISMA-P) 2015 statement^[7]^ and the Cochrane Handbook for Systematic Reviews of Interventions^[8]^ (CHSRI) will be carefully followed throughout the process.

### 2.2 The inclusion criteria

#### 2.2.1 Types of studies

Studies that report randomized controlled trials (RCTs) and quasi-RCTs of comparison between qigong and other treatment for NAFLD are considered for inclusion in this study. Studies involving non-RCTs, animal experiments, case reports, reviews will be excluded.

#### 2.2.2 Types of participants

We will include patients with NAFLD irrespective of gender, race, age, and setting. We excluded patients with any signs of mental illness or organic disease.

#### 2.2.3 Types of interventions

Using Qigong exercises for NAFLD patients. Included variation in intensity, frequency and duration will be accepted.

#### 2.2.4 Types of comparator (s)/control

Conventional treatment or exercise according to relevant guideline, or other forms of Qigong such as Tai Chi, Baduanjin, etc. Studies that compared Qigong plus another therapy with the same another therapy alone will be tolerated.

#### 2.2.5 Types of outcomes

The main outcomes are Alanine aminotransferase (ALT) (U/l) and Aspartate aminotransferase (AST) (U/l). The secondary outcomes are disappearance of radiological steatosis, NAFLD fibrosis score, the Fibrosis-4 test, the BARD index, the AST-to-platelet ratio, the FibroMeter and the FibroTest.

### 2.3 Collection and analysis of data

#### 2.3.1 Search strategy

We will perform a comprehensive literature search from the following resources: electronic databases including PUBMED, Web of Science, the Cochrane Library, Embase, SinoMed, Chinese Science and Technology Periodicals Database, Chinese National Knowledge Infrastructure (CNKI), and Wanfang Database for papers published from inception to September 2020. We will search manually for additional studies by cross-checking the reference lists of all included primary studies and lists of relevant systematic reviews. Grey literature sources including International clinical trials registry platform and 2 Qigong associations (China association of medical Qigong: http://www.cmqg.cn/, World Academic Society of Medical Qigong: http://www.wasmq88.com/sy) will be contacted for recent or unpublished papers. The search strategy will be developed by the research team in collaboration with an experienced librarian and checked by a referee according to the Peer Review of Electronic Search Strategy guidelines. The detailed information of electronic search is listed in Table 1.

**Table 1:**
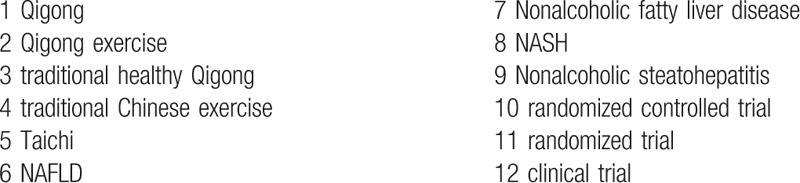
Search tactics for the PUBMED and web of science.

#### 2.3.2 Selection and extraction of data

All of the authors will be trained with Preferred Reporting Items for Systematic Review and Meta-Analysis Protocols (PRISMA-P) and CHSRI from the beginning. Review process will be undertaken by 2 reviewers (Liu and Zou) independently, applying blinding to reduce bias. Endnote V.X9 will be used to manage literature and remove duplications. The title and abstract of each article will be screened and assessed against predefined inclusion criteria. All repetitions and studies not met the inclusion criteria will be excluded. Then, they will cross-check their included and excluded studies. Study with uncertainty will be presented to the third author (Zhang) or the panel to judge if they are eligible for this study.

**Figure 1:**
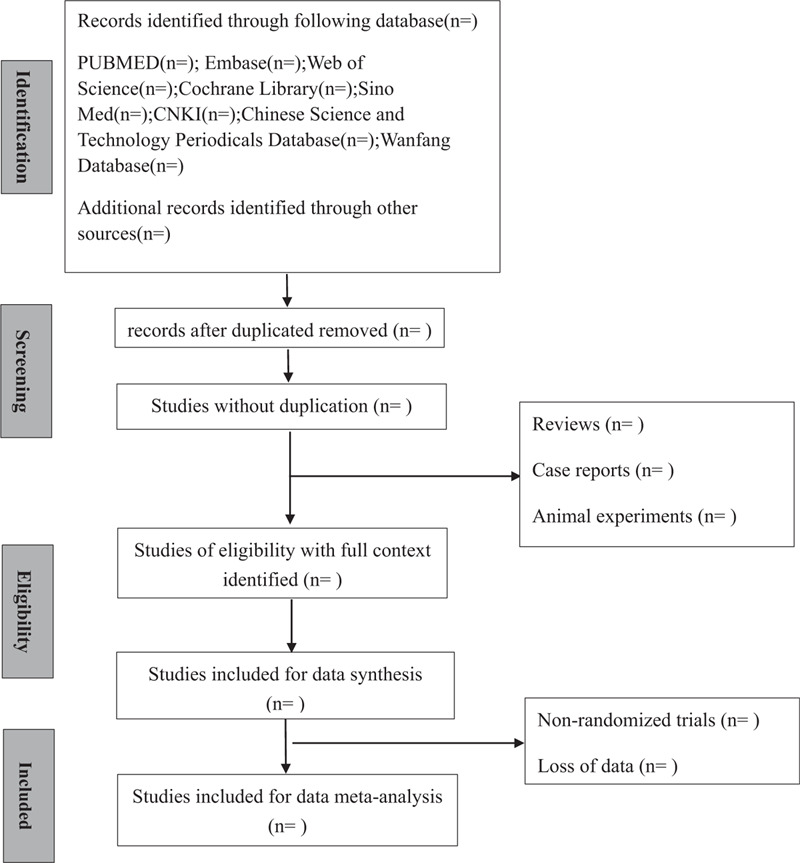
Flow diagram of studies selection process.

The standard data extraction table will be determined before data extraction. Author Liu and Zou will independently extract data in terms of general information, method description, participant and intervention, outcomes and measures and annotations of selected data. When there is uncertain study, we will have expert discussion to make a final decision. When necessary, we will contact with the authors for more related information and clinical data. Studies with no access to the original paper will be excluded. When study contains multiple groups, we will only extract data meet the inclusion criteria of this study. The diagram of this study is shown in Figure 1.

#### 2.3.3 Assessment of risk of bias in included studies

Research may vary considerably in methodological, and flaws in the design or conduct of a study can cause bias, obscuring the benefit/harm of an intervention. The methodological quality of included RCTs will be assessed according to the Cochrane Handbook for Systematic Reviews of Interventions. Since it covers the assessment of several sources of bias, including random sequence generation, allocation concealment, blinding of outcome assessments, incomplete outcome data, and selective outcome reporting. The 2 reviewers will use the Cochrane Collaboration tool to evaluate the quality of the included trials. The trial will be rated for every aspect of high, low risk, or ambiguous bias. Trials rated high risk of bias in 1 or more areas will be rated high risk, while trials rated low risk of bias in all aspects will be rated low risk. If there is an unclear risk of bias in all major areas, the trial will be rated as an unclear risk. The scoring results will be checked repeatedly and the differences will be resolved through the discussion of all reviewers.

### 2.4 Statistical analysis

#### 2.4.1 Assessment of heterogeneity

We will evaluate clinical heterogeneity first. Clinical heterogeneity refers to the variation caused by different participants, interventions and different end-point indicators of the study. Statistical heterogeneity will then evaluated using the Cochran *Q* and *I*^2^ test if there is no clinical heterogeneity. For the *I*^2^ statistic, *I*^2^ thresholds of <25%, 25% to 49%, 50% to 75% and >75% to represent low, moderate, high and very high heterogeneity respectively.

#### 2.4.2 Synthesis of data

When *I*^2^ < 75% comes from the heterogeneity test, the data will be synthesized and analyzed. When the heterogeneity test shows slight or no statistical heterogeneity in these trials (*I*^2^ value is not less than 40%), we will use a fixed-effects model for the combined data. When significant heterogeneity is detected (*I*^2^ 40%, <75%), a random effects model will be used for data synthesis. If there is considerable heterogeneity in the trial, no meta-analysis will be performed. Alanine aminotransferase (ALT) and Aspartate aminotransferase (AST) are continuous outcomes, so the mean difference or the standardized mean difference are used for meta-analysis. All the results for risk ratios are displayed in the form of forest plots. If there is considerable heterogeneity in the trial, no meta-analysis will be performed. In this case, we will try to determine the source of heterogeneity from both clinical and methodological aspects and will provide a qualitative summary.

#### 2.4.3 Additional analyses

We will perform sensitivity analysis, meta-regression and subgroup analysis based on various study characteristics and sample size, such as study type, study quality, adjustment (or not) for confounders. A brief qualitative analysis of the evidence will be presented in narrative form if data extraction is insufficient or significant differences in study methods exist.

#### 2.4.4 Assessment of reporting bias

When more than 10 trials are included, a funnel chart will be generated to observe the report deviation.

### 2.5 Quality of evidence

We will use the Grading of Recommendations Assessment, Development and Evaluation system (GRADE) system to assess the quality of evidence for each outcome. According to the GRADE rating standards, four levels of evidence quality (high, moderate, low, or very low) will be used. The quality of the included evidence will be assessed by 2 reviewers independently according to GRADE approach. Reviewers will take into account limitations of the study, inconsistencies, indirect evidence, inaccuracies, and publication bias.^[9]^

### 2.6 Ethics and dissemination

Ethical approval is not required in this study, for no clinical trials or animal experiments are involved here. Our findings will provide information about the treatment efficacy of Qigong for NAFLD patients. The establishment of this study may be published in peer-reviewed journals.

## 3 Discussion

The prevalence of nonalcoholic liver disease (NAFLD) is increasing worldwide in conjunction with the epidemic increase in obesity and metabolic risk factors.^[10]^ Treatment of patients with NAFLD is based first on dietary and lifestyle changes which should aim at reducing overweight and reducing or controlling pathological conditions associated with overweight. Lack of physical exercise are associated with metabolic syndrome and NAFLD, leading to an epidemic in disease prevalence globally.^[11]^ Based on previous research, the effects of exercise may ameliorate NAFLD include: increasing caloric consumption and facilitating weight loss, reducing intrahepatic fat/triacylglycerol, even in the absence of weight loss, and increasing insulin sensitivity.^[12]^ But there is no consensus on the type of exercise that should be recommended to patients with NAFLD.

The liver is in charge of storing the blood and maintaining a free flow of qi (vital energy) throughout all of the organs.^[13]^ Improper lifestyle habits such as lack of exercise affects the function of the liver. Liver qi stagnation is a common pattern in NAFLD since there is a build-up of fat in the liver. Qigong is practiced by Chinese people to improve health, explore the latent ability of humans, prevent disease and prolong life in the context of a wide range of conditions,^[14]^ including metabolic syndrome,^[15]^ diabetes,^[16]^ stress,^[17]^ anxiety,^[18]^ fatigue,^[19]^ hypertension,^[20]^ heart failure,^[21]^ immune function,^[22]^ and to enhance the quality of life of patients with other chronic diseases.^[23]^ Nowadays, Qigong is increasing in popularity among the general population in Western countries. In this study, we will review all related clinical reports and analyze the clinical data to evaluate the safety and effectiveness of these therapies and to provide solid evidence for potential treatment of Qigong for NAFLD in future utility. We will try to thoroughly search the electronic databases, clinical online registration websites and get in touch with authors of included studies for more detailed information to get a complete data as possible. The results of this review may help to establish a better approach to treat the disease and suspend the deterioration of liver function and provide rigorous evidence for its clinical application and dissemination.

## Author contributions

**Conceptualization:** Yuqiao Liu.

**Data curation:** Yuqiao Liu, Jiaxi Zou.

**Formal analysis:** Lijuan Dan.

**Software:** Lijuan Dan.

**Supervision:** Renyan Zhang.

**Writing – original draft:** Yuqiao Liu.

**Writing – review & editing:** Quansheng Feng.
